# *Spiroplasma* infection as a cause of severe congenital keratouveitis, cataract and glaucoma

**DOI:** 10.1186/s12886-024-03480-z

**Published:** 2024-05-21

**Authors:** Helena Van Haecke, Dimitri Roels, Fanny Nerinckx, Heidi Schaballie, Petra Schelstraete, Linos Vandekerckhove, Jolien Van Cleemput, Wim Van den Broeck, Liesbeth Couck, Hannelore Hamerlinck, Stien Vandendriessche, Jerina Boelens, Inge Joniau

**Affiliations:** 1 Department of Ophthalmology, Ghent University Hospital, Corneel Heymanslaan 10, Ghent, 9000 Belgium; 2 Department of Pediatric Infectious Diseases, Ghent University Hospital, Ghent, Belgium; 3 HIV Cure Research Center, Dpt of Internal Medicine and Pediatrics, Ghent University, Ghent, Belgium; 4 Department of Morphology, Imaging, Orthopedics, Rehabilitation and Nutrition, Ghent University, Ghent, Belgium; 5 Department of Medical Microbiology, Ghent University Hospital, Ghent, Belgium

**Keywords:** Congenital keratouveitis, Cataract, Angle-closure glaucoma, *Spiroplasma ixodetis*

## Abstract

**Background:**

Only seven cases of ocular *Spiroplasma* infection have been reported to date, all presenting as congenital cataracts with concomitant intraocular inflammation. We describe the first case of *Spiroplasma* infection initially presenting as a corneal infiltrate.

**Case presentation:**

A 1-month-old girl was referred for a corneal infiltrate in the left eye. She presented in our hospital with unilateral keratouveitis. Examination showed a stromal corneal infiltrate and dense white keratic precipitates in the left eye. Herpetic keratouveitis was suspected and intravenous acyclovir therapy was initiated. Two weeks later, the inflammation in the left eye persisted and was also noticed in the right eye. Acute angle-closure glaucoma and a cataract with dilated iris vessels extending onto the anterior lens capsule developed in the left eye. The inflammation resolved after treatment with azithromycin. Iridectomy, synechiolysis and lensectomy were performed. Bacterial metagenomic sequencing (*16 S rRNA*) and transmission electron microscopy revealed *Spiroplasma ixodetis* species in lens aspirates and biopsy. Consequently, a diagnosis of bilateral *Spiroplasma* uveitis was made.

**Conclusions:**

In cases of congenital cataract with concomitant intraocular inflammation, *Spiroplasma* infection should be considered. The purpose of this case report is to raise awareness of congenital *Spiroplasma* infection as a cause of severe keratouveitis, cataract and angle-closure glaucoma in newborns. Performing molecular testing on lens aspirates is essential to confirm diagnosis. Systemic macrolides are suggested as the mainstay of treatment.

## Background

*Spiroplasma* is a genus of bacteria classified within the class Mollicutes, characterized by the lack of a cell wall. Until now, only seven cases of ocular *Spiroplasma* infection have been described, all presenting as congenital cataracts with concomitant intraocular inflammation. We add one more case to raise awareness of congenital *Spiroplasma* infection with ophthalmic complications. It is the first report of a *Spiroplasma* infection presenting as a corneal infiltrate, with rapid evolution towards bilateral anterior uveitis and cataract.

## Case presentation

**Fig. 1:**
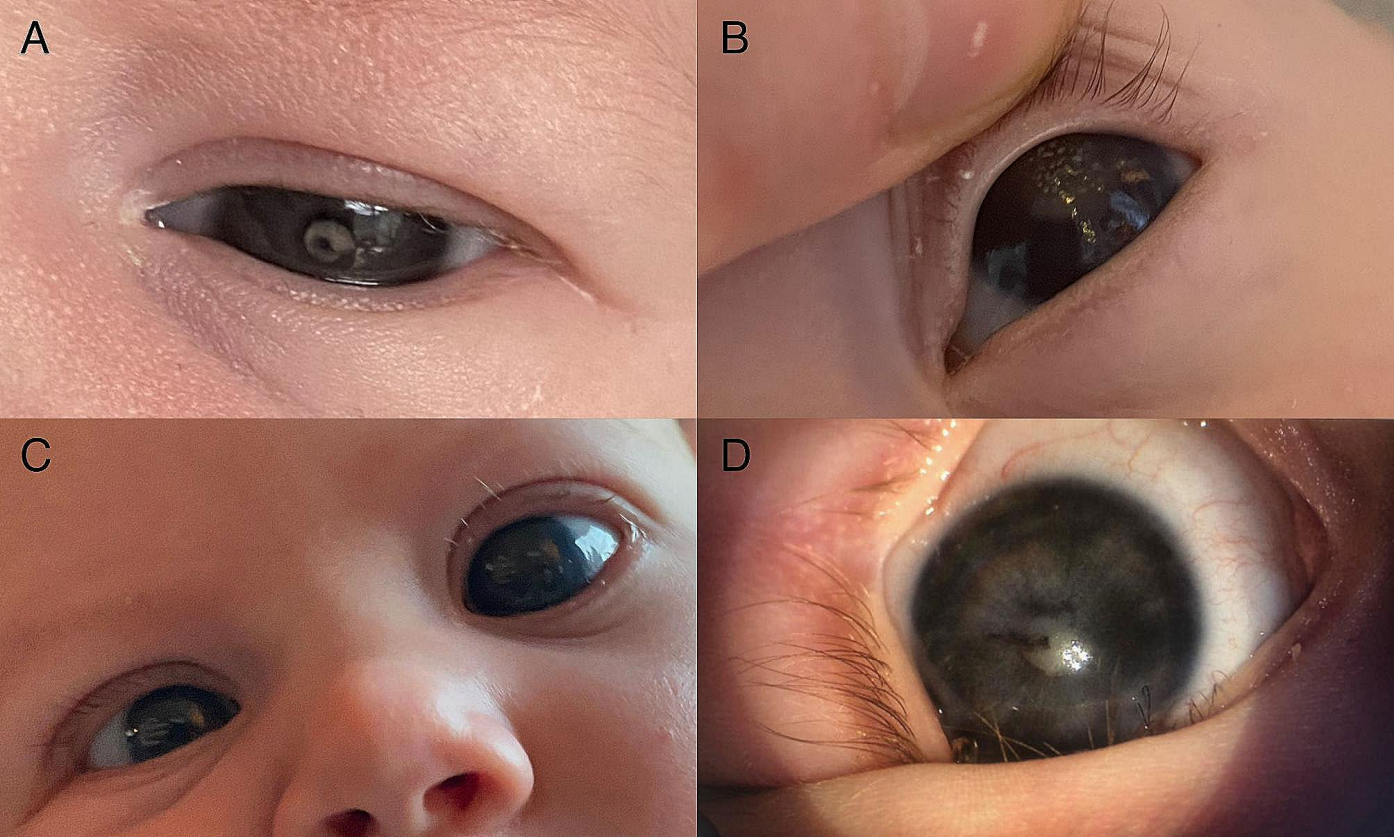
Clinical photography. (**A**) Picture of the left eye showing C-shaped white corneal stromal infiltrate. (**B**) Notice keratic precipitates in the left eye. (**C**) Picture showing buphthalmos of the left eye. (**D**) Intraoperative picture of the left eye after one week of treatment with oral azithromycin, notice regression of keratic precipitates and development of white cataract

A 1-month-old infant was referred for a corneal infiltrate in the left eye. She was a healthy term infant. Her parents had already noticed blurring of the left eye from the age of 1 week. In retrospect, a C-shaped white corneal infiltrate was visible in the parent’s photographs (Fig. 1A). Slit lamp examination showed central corneal epitheliopathy, stromal oedema and white endothelial precipitates in the left eye. Herpetic keratouveitis in the left eye was suspected. She was admitted to a primary care hospital for intravenous acyclovir administration. Serologic results for *Toxoplasma gondii (IgA, IgM, IgG)*, *Toxocara (IgG)*, *Leptospira (combined IgM/IgG)*, syphilis (treponemal and non-treponemal test), zika virus (IgM, IgG), cytomegalovirus (CMV) (IgM, IgG), herpes simplex virus (HSV) (IgM, IgG), varicella zoster virus (VZV) (IgM, IgG) and human immunodeficiency virus (p24 antigen and HIV-1 and HIV-2 antibodies) were negative.

Despite treatment, 2 weeks later, the girl developed angle-closure glaucoma and buphthalmia with increased keratic precipitates in the left eye and a prepupillary membrane in the right eye (Fig. 1B and C). Acyclovir was stopped and she was admitted to our hospital for further investigation. Treatment with topical prednisolone 1%, dorzolamide/timolol, latanoprost and tropicamide was initiated. Systemic intraocular pressure (IOP) lowering therapy was administered.

**Fig. 2:**
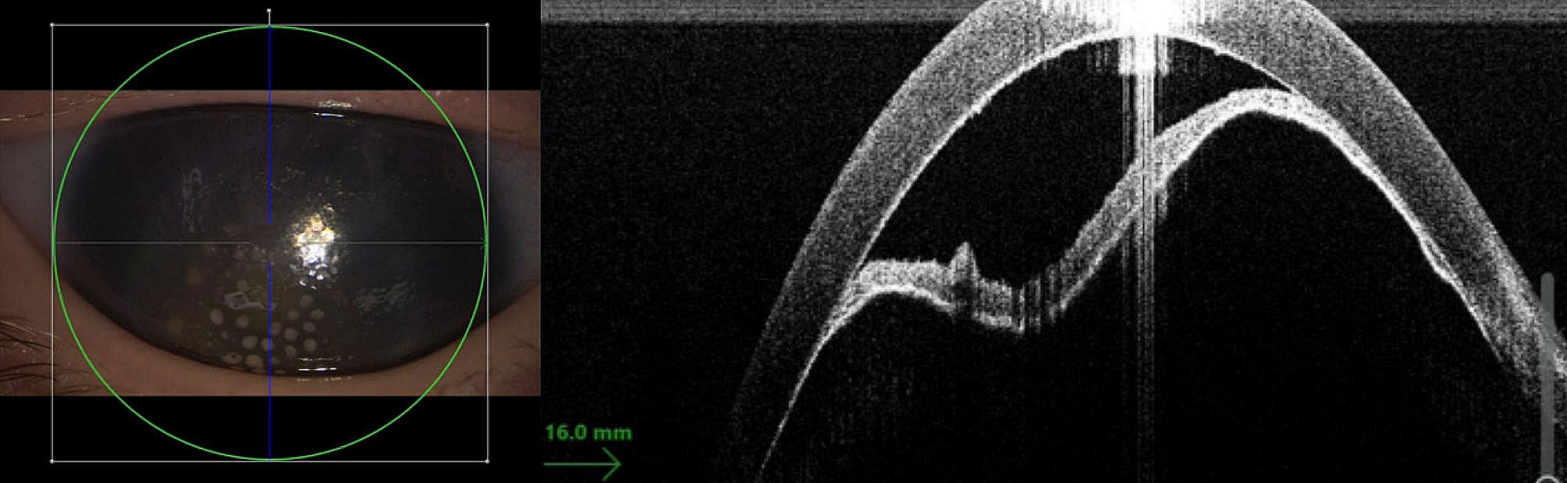
Intraoperative anterior segment optical coherence tomography of the left eye. Notice keratic precipitates and complete angle closure with iris bombe

Examination under general anaesthesia revealed bilateral anterior uveitis with posterior synechiae and cataract. In the left eye, there was 360° of iridocorneal apposition (Fig. 2) and a white cataract with dilated iris vessels extending from the pupillary margin onto the anterior lens capsule. The fundus was not visible in either eye. The IOP of the left eye was 25 mmHg (right eye 13.5 mmHg). Buphthalmos of the left eye was confirmed by ultrasound imaging. The axial length of the left eye was 21 mm (right eye 18 mm). The corneal diameter of the left eye was 12 mm (right eye 9.5 mm). The infant underwent a bilateral anterior chamber tap and a posterior synechiolysis. In addition, an iridectomy was performed to resolve the pupillary block in the left eye. Cefuroxime was injected intracamerally. Empirical broad spectrum antimicrobial therapy was initiated with oral trimethoprim/sulfamethoxazole and azithromycin. Intravenous prednisolone (2 mg/kg/d for 3 days) was added to the therapeutic regimen.

Inflammation decreased after one week of treatment (Fig. 1D). A lensectomy was performed sequentially in both eyes (left, then right, approximately two months apart). Given the presence of vascularization onto the lens capsule, intravitreal anti-VEGF was administered. Intraoperatively, a chorioretinal lesion in the posterior pole of the left eye was observed.

**Fig. 3:**
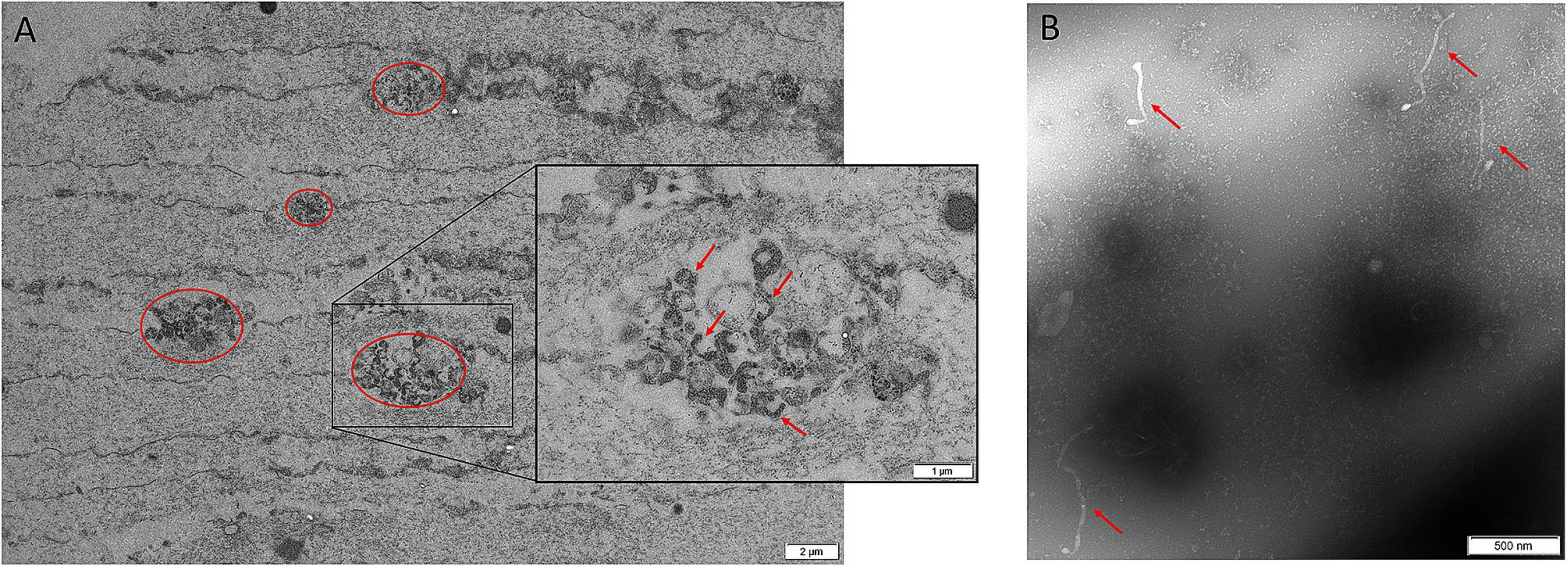
Transmission electron microscopy images showing Spiroplasma sp. (**A**) The red ovals highlight focal aggregations of Spiroplasma sp. in lens biopsy sections. The red arrows in the higher magnification show multiple filamentous, curved, round, and helical profiles of Spiroplasma sp. (**B**) Morphological forms of Spiroplasma sp. isolated from anterior chamber fluid (red arrows)

Polymerase chain reaction (PCR) assays done on anterior chamber fluid were negative for CMV, HSV-1, HSV-2, VZV, Epstein-Barr virus, human herpesvirus 6 and 7. A panbacterial 16 S ribosomal RNA PCR on anterior chamber fluid was negative. Transmission electron microscopy of lens biopsy sections of the left eye showed the presence of *Spiroplasma* species (Fig. 3). Metagenomic sequencing targeting the complete 16 S rRNA gene performed on aspirates of the right lens identified *Spiroplasma ixodetis*.

Postoperatively, the infant received topical tobramycin, prednisolone 1%, dorzolamide/timolol and latanoprost. Treatment with oral azithromycin was continued for a total of 16 weeks. Intraocular inflammation did not recur postoperatively. Six weeks after cataract surgery, we observed repeated episodes of IOP rise in the left eye considered to be caused by anterior synechiae. The IOP normalized after laser cyclophotocoagulation. Topical timolol, dorzolamide and latanoprost were continued. At last follow up, 5 months after lensectomy in the left eye and 3.5 months after lensectomy in the right eye, IOP was 17mmHg in the right eye and 20mmHg in the left eye. Binocular visual acuity with aphakic spectacles was 2.4 cycles per cm (Teller Acuity Cards at 38 cm).

## Discussion

*Spiroplasma* is a genus of bacteria belonging to the class Mollicutes. Mollicutes lack a peptidoglycan cell wall and are enclosed in a three-layered cell membrane. These organisms utilize cytoskeletal elements for cell elongation, cell division and motility. *Spiroplasma* species are helical or spiral-shaped and have a broad host range including plants, insects, and even vertebrates. *Spiroplasma* have also been shown capable of infecting humans. Still, little is known about the transmission between animals and humans [1]. To date, systemic *Spiroplasma* infection has been reported in four immunocompromised adult patients and one immunocompetent patient [2–5]. These patients presented with arthritis, fever, myalgia, fatigue or headaches.

Lorenz et al. were the first to report a case of ocular *Spiroplasma* species infection in a premature baby, which manifested as a unilateral cataract associated with anterior uveitis [6]. To date, only six more cases of ocular *Spiroplasma* have been reported worldwide [7–9]. The timing of symptom presentation varied, with some infants showing symptoms shortly after birth and others only after a few weeks. All cases involved cataract and anterior uveitis with keratic precipitates and posterior synechiae. However, the severity and specific manifestations were diverse. In most infants, immature dilated iris vessels were present [6–8]. Two cases mentioned elevated IOP and buphthalmos [8, 9], similar to our case. Only one patient showed the presence of vitreous inflammation and retinal lesions [8] mirroring our case. In contrast to all the existing reports on ocular *Spiroplasma* infection, the patient in our study was initially referred for a unilateral corneal infiltrate. A rapid progression to bilateral anterior uveitis and cataract was seen.

The choice of treatment depended on the specific symptoms and findings in each case. Lensectomy, performed in all cases, allowed for the identification of *Spiroplasma* species. Oral macrolides (josamycin/erythromycin/azithromycin) combined with topical and/or systemic steroids resulted in rapid resolution of inflammation in all patients. Treatment with oral antibiotics was continued for a total of 10 days to 5 weeks. In our case, inflammation decreased after one week of treatment with oral azithromycin. However, the therapy was continued for a total of 16 weeks due to a two-month period between the lensectomy of the left and right eye respectively.

Additional treatment was required in the presence of elevated IOP. In one infant, topical steroids and β-blockers in combination with oral josamycin allowed for the normalization of IOP within a day and for the resolution of inflammation within 2 weeks [9]. In the other case with reported IOP rise, a 5-week course of a topical and systemic anti-inflammatory, antibiotic and IOP-lowering therapeutic regimen was administered [8]. Additionally, in our case, the infant required laser cyclophotocoagulation after iridectomy and lensectomy to normalize IOP.

## Conclusion

We present the first case of ocular *Spiroplasma* infection initially presenting as a corneal infiltrate mimicking herpetic keratouveitis. The purpose of this case report is to raise awareness of congenital *Spiroplasma* infection as a cause of severe keratouveitis, cataract and angle-closure glaucoma in newborns. Performing bacterial metagenomic sequencing on lens aspirates is essential to confirm diagnosis. Systemic macrolides are suggested as the mainstay of treatment. Further research is needed to develop treatment guidelines.

## Data Availability

The data supporting our findings is presented within the manuscript.

## Abbreviations

IOP: intraocular pressure
PCR: polymerase chain reaction
CMV: cytomegalovirus
HSV: herpes simplex virus
VZV: varicella zoster virus
